# Anlotinib affects systemic lipid metabolism and induces lipid accumulation in human lung cancer cells

**DOI:** 10.1186/s12944-023-01907-y

**Published:** 2023-08-23

**Authors:** Zhongling Zhu, Shan Xu, Jing Ren, Teng Jiang, Cai Zhang, Zhao Yan

**Affiliations:** 1 Department of Clinical Pharmacology, Tianjin’s Clinical Research Center for Cancer, Key Laboratory of Cancer Prevention and Therapy, Tianjin Medical University Cancer Institute & Hospital, National Clinical Research Center for Cancer, Tianjin, China; 2 Department of Continuing Education and Science and Technology Service, China Anti-Cancer Association, Tianjin, China

**Keywords:** Anlotinib, Hyperlipidemia, Non-small cell lung cancer, Lipid metabolism, Low-density lipoprotein receptor

## Abstract

**Background:**

Anlotinib has demonstrated encouraging clinical outcomes in the treatment of lung cancer, soft tissue sarcoma and thyroid carcinoma. Several clinical studies have shown a relationship between anlotinib treatment and the occurrence of hyperlipidemia. The fundamental mechanisms, however, are still largely unclear. Here, the effect of anlotinib on lipid metabolism in an animal model and human cancer cells was evaluated and the role of lipid metabolism in the antitumor efficacy of anlotinib was investigated.

**Methods:**

The C57BL/6 J mouse model as well as A549 and H460 human lung cancer cell lines were used to examine the impact of anlotinib on lipid metabolism both in vivo and in vitro. Levels of triglycerides, high-density lipoprotein, low-density lipoprotein (LDL), and total cholesterol in serum or cell samples were determined using assay kits. The expression levels of crucial genes and proteins involved in lipid metabolism were measured by quantitative RT-PCR and Western blotting. Furthermore, exogenous LDL and knockdown of low-density lipoprotein receptor (LDLR) were used in H460 cells to investigate the relevance of lipid metabolism in the anticancer efficacy of anlotinib.

**Results:**

Anlotinib caused hyperlipidemia in C57BL/6 J mice, possibly by downregulating hepatic LDLR-mediated uptake of LDL cholesterol. AMP-activated protein kinase and mammalian target of rapamycin inhibition may also be involved. Additionally, anlotinib enhanced sterol response element binding protein 1/2 nuclear accumulation as well as upregulated LDLR expression in A549 and H460 cells, which may be attributable to intracellular lipid accumulation. Knockdown of LDLR reduced intracellular cholesterol content, but interestingly, anlotinib significantly improved intracellular cholesterol accumulation in LDLR-knockdown cells. Both exogenous LDL and LDLR knockdown decreased the sensitivity of cells to anlotinib.

**Conclusions:**

Anlotinib modulates host lipid metabolism through multiple pathways. Anlotinib also exerts a significant impact on lipid metabolism in cancer cells by regulating key transcription factors and metabolic enzymes. In addition, these findings suggest lipid metabolism is implicated in anlotinib sensitivity.

**Supplementary Information:**

The online version contains supplementary material available at 10.1186/s12944-023-01907-y.

## Introduction

Hyperlipidemia is characterized by abnormally high blood lipid and/or lipoprotein levels. It is well acknowledged as a significant risk factor related to cancer, diabetes, and cardiovascular disease [1–4].

Anlotinib is an innovative multitargeted tyrosine kinase inhibitor (TKI) that effectively targets various receptor tyrosine kinases implicated in tumor angiogenesis as well as growth. Results from the ALTER 0303 trial demonstrated that anlotinib exhibited notable clinical efficacy and good tolerability, even in patients with advanced non-small cell lung cancer who have had at least two lines of systemic treatment and have suffered disease progression or recurrence [5]. Interestingly, the incidence of hyperlipidemia was found to be significantly greater in anlotinib-treated patients compared to placebo-treated patients. In the anlotinib group, 44.6% of patients experienced hypertriglyceridemia, whereas in the placebo group, the incidence was 23.8%. Similarly, the incidence of hypercholesterolemia was also higher in the anlotinib group (41.8%) than in the placebo group (14.0%). Furthermore, anlotinib treatment was associated with low-density lipoprotein (LDL) elevation in 21.1% of patients, whereas the incidence in the placebo group was 7.7%. Wang et al. [6] reported that hypertriglyceridemia and hypercholesterolemia occurring during treatment were correlated with longer progression-free survival (PFS) and overall survival (OS). Additionally, hypertriglyceridemia was identified as an independent predictor for both PFS and OS. The incidence of increased triglyceride and hypercholesterolemia in the anlotinib group was 21.4% in a recent study on metastatic renal cell cancer (mRCC) (NCT02072044) [7]. Another study (NCT02072031) suggested that anlotinib had a tendency to have a greater prevalence of hypercholesterolemia in comparison to sunitinib [8]. However, the precise molecular mechanisms underlying anlotinib-induced hyperlipidemia remain largely unknown.

Anticancer drugs that inhibit phosphoinositide 3-kinase (PI3K)/Akt/mammalian target of rapamycin (mTOR) signaling has been discovered to induce hyperlipidemia [9–12]. Additionally, increasing evidence has demonstrated that metabolic effects such as hyperlipidemia and hypothyroidism are common adverse events associated with TKIs, such as famitinib, pazopanib, sorafenib and sunitinib [13–16]. Preliminary data have showed that synergistic interactions between platelet-derived growth factor (PDGF) and LDL-related protein (LRP) indicates a potential impact on lipid metabolism [17, 18]. Since PDGF receptor is one of the main targets of sunitinib, some studies have supposed that sunitinib induces hyperlipidemia through PDGF and LRP signaling. In addition, hypothyroidism can lead to hyperlipidemia by downregulating LDL receptor (LDLR) in the liver, thereby reducing LDL clearance. Thyroid function may be one of the factors influencing lipid metabolism.

The purpose of this study was to examine how anlotinib affects lipid metabolism in a mouse model and cancer cell lines, and to explore the fundamental mechanisms through which anlotinib impacts on lipid metabolism. These findings shed light on the higher prevalence of hyperlipidemia in anlotinib-treated patients and the involvement of lipid metabolism in the antitumor efficacy of anlotinib.

## Methods

### Reagents

Anlotinib was kindly provided by Chia Tai Tian Qing Pharmaceutical Group Co., Ltd (Nanjing, China). Low-density lipoprotein (LDL) and HPLC grade rapamycin were obtained from Merck (Darmstadt, Germany). The COD-PAP kit for total cholesterol (TC) concentration, the GPO-PAP kit for triglyceride (TG) concentration, the high-density lipoprotein cholesterol (HDL-c) colorimetric assay kit, and the low-density lipoprotein cholesterol (LDL-c) colorimetric assay kit were purchased from Jiancheng Bioengineering Institute (Nanjing, China). The Oil Red O staining kit was purchased from Beijing Solarbio Science & Technology Co., Ltd. (Beijing, China). Antibodies against AMP-activated protein kinase (AMPK) (#5832), phospho-AMPK (Thr172) (#2535), mTOR (#2983), phospho-mTOR (Ser2448) (#5536) were obtained from Cell Signaling Technology (Danvers, MA, USA). Antibodies against 3-hydroxy-3-methylglutaryl-coenzyme A reductase (HMGCR) (ab242315), LDLR (ab52818) were purchased from Abcam (Cambridge, MA, USA). Antibodies against sterol response element binding protein 1 (SREBP1) (sc-13551), sterol response element binding protein 2 (SREBP-2) (sc-13552) and GAPDH (sc-32233) were purchased from Santa Cruz Biotechnology (Santa Cruz, CA, USA). IRDye-conjugated anti-rabbit or anti-mouse IgG secondary antibodies were obtained from LI-COR Biosciences (Lincoln, NE, USA). PVDF membranes were purchased by Millipore Corporation (Bedford, MA, USA) and the bicinchoninic acid (BCA) assay was obtained by Pierce Biotechnology, Inc., (Rockford, IL, USA). TRIzol reagent was purchased from Invitrogen Inc., (Carlsbad, CA, USA). The RevertAid First Strand cDNA Synthesis Kit was purchased from Fermentas (Thermo Fisher Scientific, Inc.) and the SYBR® Green Real-time PCR Master Mix was purchased from Toyobo Co., Ltd., (Tokyo, Japan). Dulbecco’s modified Eagle’s medium (DMEM), fetal bovine serum (FBS), trypsin, and penicillin/streptomycin were purchased from Invitrogen (Carlsbad, CA, United States). 3- (4,5-dimethylthiazol-2-yl)-2,5-diphenyltetrazolium bromide (MTT), dimethyl sulfoxide (DMSO), phosphate-buffered saline (PBS), bovine serum albumin (BSA), TBST buffer, paraformaldehyde and hematoxylin–eosin (H&E) staining were purchased from Solarbio Bioscience & Technology Co. Ltd (Beijing, China).

### Animals / mice

Female C57BL/6 J mice aged four weeks were purchased from HFK Bioscience Co., Ltd (Beijing, China). The mice were kept in a controlled environment with a temperature range of 22–28 °C, a relative humidity range of 60–70%, and a 12-h cycle of darkness and light. Following a 3-day adaption period, the mice were randomly allocated to three groups: control group (treated with vehicle, 0.2% carboxymethylcellulose), anlotinib group (3 mg/kg) and rapamycin group (2 mg/kg). Anlotinib and rapamycin were administered intragastrically once daily. Each mouse's body weight was recorded once a week. Following 21 days of treatment, the mice had a 12-h fast before having blood drawn from the eyes. Centrifugation at 3500 rpm for 5 min at 4 °C yielded serum, which was kept at -80 °C. By cervical dislocation, the mice were euthanized and liver tissue was collected. For sectioning and H&E staining, a part of the liver tissue was preserved in 4% paraformaldehyde. The remainder of the liver was frozen in liquid nitrogen promptly. The Laboratory Animal Ethics Committee of Tianjin Medical University Cancer Institute and Hospital examined and approved the animal study protocol.

### Cell culture

A549 and H460 cells were obtained from the American Type Culture Collection (Manassas, VA, USA) and validated by DNA sequencing on an ABI 3730xl genetic analyzer. HEK 293 T cells were acquired from the Type Culture Collection of the Chinese Academy of Sciences (Shanghai, China). The cells were grown in DMEM containing 10% FBS, 100 units/ml penicillin, and 100 μg/ml streptomycin. All the cells were cultured in a humidified atmosphere with 5% CO_2_ at 37 °C.

### MTT cell viability assay

In 96-well plates, cells were planted at a density of 4 × 10^3^ per well. Various concentrations of anlotinib (0.625–20 μM) or PBS (vehicle) were administered to the cells after 24 h. Following a 72-h incubation, each well added 20 μl of 5 mg/mL MTT solution and was incubated for 4 h. After carefully aspirating the supernatant, 150 μl of DMSO was used to dissolve the formazan crystals. With the use of a microplate reader (Epoch2, Biotek), the absorbance was measured at 490 nm.

### Lentivirus preparation and cell infection

Short hairpin RNAs (shRNAs) targeting LDLR gene were synthesized by GENEWIZ, Inc. (Suzhou, China). The following were the precise sequences used: LDLR sequence 1 (GATGAAGTTGGCTGCGTTAATCTCGAGATTAACGCAGCCAACTTCATC, #1), LDLR sequence 2 (ATGGAAGAACTGGCGGCTTAACTCGAGTTAAGCCGCCAGTTCTTCCAT, #2) and a scramble non-specific control sequence (CCTAAGGTTAAGTCGCCCTCGCTCGAGCGAGGGCGACTTAACCTTAGG). The shRNAs were then cloned into the PLKO.1 vector using AgeI/EcoRI restriction sites, resulting in recombinant lentiviral shRNA expression vectors. HEK 293 T cells were co-transfected with VSV-G, △8.91 and the shRNA expression plasmids using the transfection agent polyethylenimine. After 48 h, the lentiviral particle-containing supernatants were collected and used to infect H460 cells while containing 10 g/mL polybrene. In the presence of 2 μg/mL puromycin, stable cell clones with scramble or LDLR knockdown were selected.

### Wound-healing assay

At a density of 2 × 10^4^ cells per well, cells were planted in a 6-well plate and cultured until reaching confluence. A sterile 200 µL pipette tip was used to make a straight-line scratch wound. The cells were then gently rinsed with PBS buffer to eliminate cellular debris. Following that, the cells were cultured for 48 h at 37 °C with either vehicle or anlotinib. After 0 h, 24 h, and 48 h, phase contrast pictures of the wounds were obtained and the indicated time points were used to assess the wound's width. Using Image J software (National Institutes of Health, NIH), the wound area was measured at 0 h, as well as the healing areas at 24 and 48 h. The following formula was used to determine the migration rate: migration rate % = 100% - (area time n hours/area time 0 h × 100%).

### RNA extraction and qRT-PCR analysis

The TRIzol reagent was used to extract total RNA from tumor cells or liver tissues. Following the manufacturer's instructions, 1 g of total RNA was reverse transcribed into cDNA using the RevertAid first strand cDNA synthesis kit. On a CFX96 Real-Time PCR Detection System (Bio-Rad), real-time qPCR was performed using SYBR® Green Realtime PCR Master Mix. GAPDH was used as the internal control gene and the expression data were normalized to 18S rRNA expression. The primer sequences utilized in this investigation were produced by Aoke Dingsheng Biotechnology Co., Ltd (Beijing, China) (Tables S1 and S2).

### Western blot analysis

The cells were lysed using SDS lysis buffer after being rinsed with ice-cold PBS. A mortar and pestle were used to crush frozen liver tissues, which were subsequently incubated in lysis buffer overnight at 4 °C. The lysates were collected by centrifugation at 4 ℃, 10,000 g for 10 min. The BCA assay was used to determine the total protein content. SDS–polyacrylamide gels were used to separate the proteins, which were then wet electrotransferred onto PVDF membranes. After blocking the membranes for an hour at room temperature with 5% BSA in TBST buffer, the primary antibodies were incubated on the membranes overnight at 4 °C. The membranes were then incubated for an hour at room temperature with IRDye-conjugated anti-rabbit or anti-mouse secondary antibodies after being rinsed three times with TBST buffer for 10 min each. The membranes were visualized using an Odyssey infrared imaging system after three further TBST washing processes.

### Determination of serum lipids

According to the instructions provided by the manufacturer (Jiancheng Bioengineering Institute), the levels of TC, TG, LDL-c, and HDL-c in serum or cell samples were determined using assay kits.

### Oil Red O staining

According to the manufacturer's instructions, Oil Red O staining was performed utilizing an assay kit. In brief, the cells were gently rinsed three times in PBS buffer before being fixated for 15 min in ORO fixative solution. The cells were then rinsed three times for 5 min each with 60% isopropyl alcohol. After 20 min of staining with a filtered Oil Red O working solution, the cells were washed five times with ddH2O. Following that, the cells were stained with Mayer hematoxylin staining solution for 2 min, washed with ddH_2_O five times, and soaked in ORO buffer for 5 min. Finally, the cells were observed and photographed using an EVOS imaging system.

### Statistical analysis

All data from three different experiments are expressed as mean ± SD and were analyzed with GraphPad Prism 8. The unpaired Student's *t*-test was used to compare the mean values. The Shapiro–Wilk test was used to determine the normality distribution. A *P* value of 0.05 was used to define statistical significance. *P* < 0.05 is represented by the symbol *, and *P* < 0.01 by the symbol **.

## Results

### Anlotinib treatment causes hyperlipidemia in C57BL/6 J mice

**Fig. 1:**
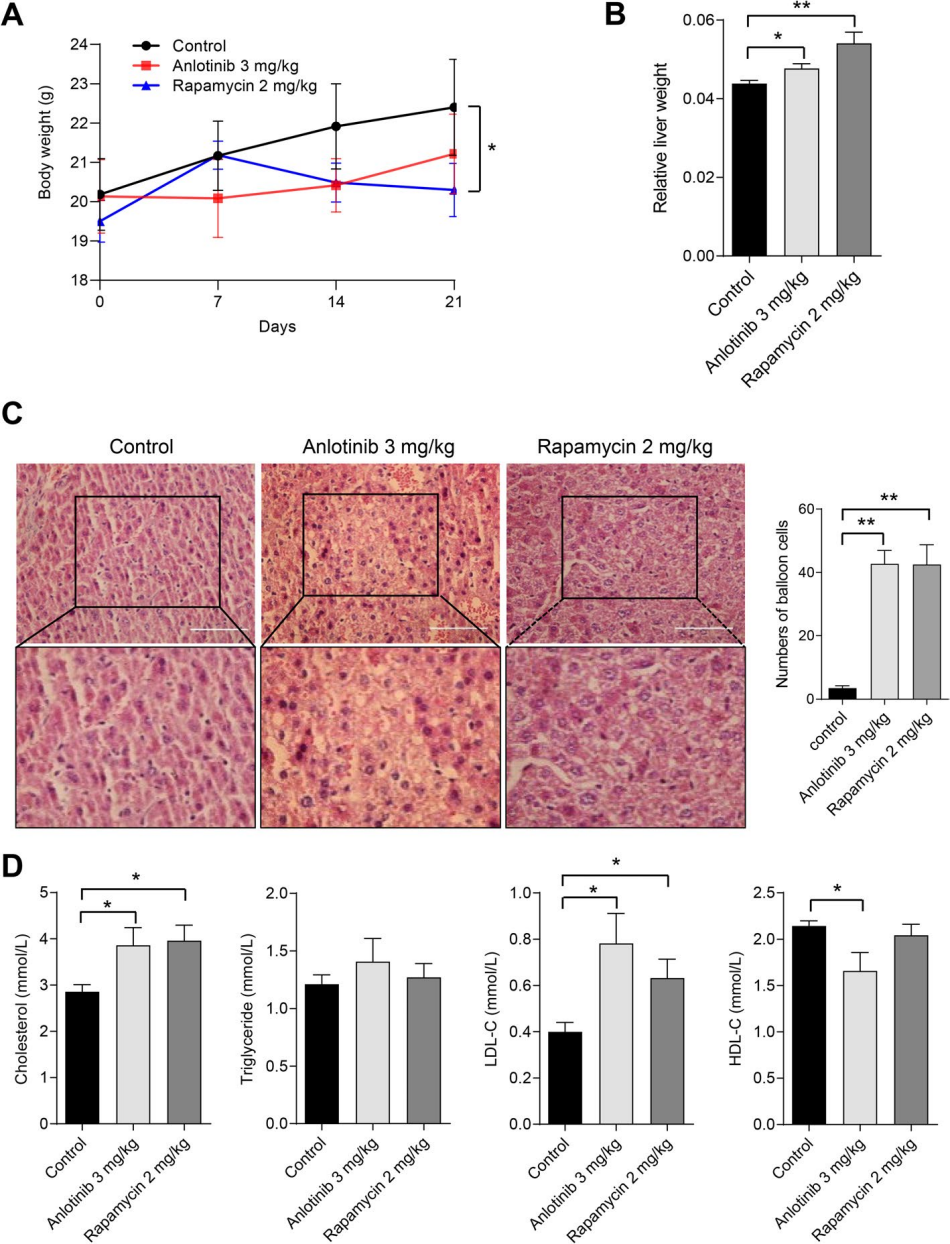
Anlotinib treatment induces hyperlipidemia in C57BL/6 J mice. According to the Materials & Methods, C57BL/6 J mice were randomly assigned to one of three groups and given three different treatments for 21 consecutive days: vehicle (circles), anlotinib (squares), and rapamycin (triangles). Variations in body weights (**A**) and relative liver weights (liver/body weight ratio) (**B**) over the course of treatment. **C** Staining of liver sections with H&E. Scale bar = 100 μm. **D** The amounts of TC, TG, LDL, and HDL in the serum. *P < 0.05; **P < 0.01 by Student’s *t* test

The body weight variations between the anlotinib group and the control group failed to approach statistical significance. On the other hand, a substantial reduction in body weights was seen after three weeks of rapamycin treatment (Fig. 1A). Furthermore, both anlotinib-treated and rapamycin-treated mice showed a notable increase in relative liver weight (Fig. 1B). H&E staining revealed histopathological changes such as hepatocellular ballooning degeneration in the groups treated with anlotinib and rapamycin (Fig. 1C). Moreover, anlotinib or rapamycin-treated mice had significantly higher blood fasting TC and LDL levels than control mice, although neither group's TG levels changed. Additionally, in the anlotinib group, HDL levels declined considerably (Fig. 1D). These results revealed that anlotinib treatment significantly affected host lipid homeostasis, notably impacting cholesterol metabolism.

### Anlotinib treatment results in various effects on crucial regulators of cholesterol pathway in the liver

**Fig. 2:**
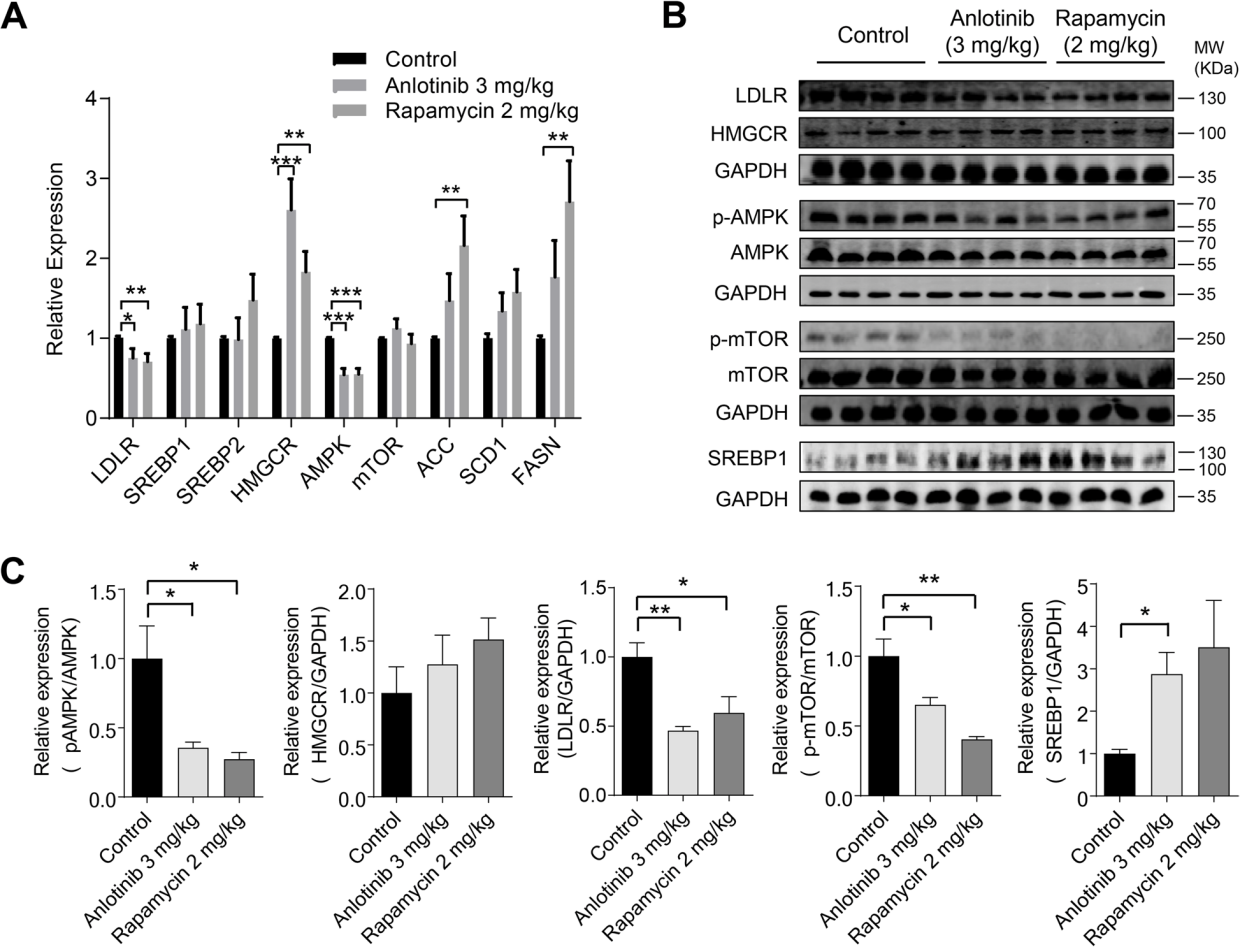
Anlotinib treatment regulates key genes and enzymes required for cholesterol synthesis and uptake in the liver. **A** LDLR, SREBP1, SREBP2, HMGCR, AMPK, mTOR, ACC, SCD1 and FASN mRNA expression levels in the liver. **B** LDLR, HMGCR, p-AMPK, AMPK, p-mTOR and mTOR protein expression levels in the liver. **C** Corresponding histograms for protein expression levels. *P < 0.05; **P < 0.01 by Student’s *t* test

Several important upstream transcriptional factors and their downstream enzymes are involved in the regulation of lipid metabolism. To gain a better understanding of how anlotinib administration leads to hypercholesterolemia and LDL elevation, the mRNA and protein levels of these transcriptional factors and enzymes were examined. HMGCR, a critical downstream gene involved in cholesterol biosynthesis, showed a considerable increase in its mRNA level following treatment with anlotinib or rapamycin. Conversely, the mRNA level of LDLR, responsible for LDL-c uptake, was greatly reduced. However, the mRNA levels of SREBP1 and SREBP2, the main upstream regulators of cholesterol, remained unaffected. Additionally, acetyl CoA carboxylase 1 (ACC1), fatty acid synthase (FASN), and stearoyl-CoA desaturase 1 (SCD1), genes involved in fatty acid synthesis, as well as mTOR, a key metabolic regulator, were not affected by anlotinib treatment. Interestingly, both the anlotinib and rapamycin groups showed a considerable reduction in the mRNA level of AMPK, a well-known regulator of lipid metabolism (Fig. 2A). Furthermore, anlotinib treatment resulted in a marked rise in SREBP1 protein expression and a decrease in phospho-AMPK, phospho-mTOR, and LDLR protein expression. However, the expression of HMGCR proteins remained unaffected by anlotinib treatment (Fig. 2B and C). These findings suggest that anlotinib treatment likely influences cholesterol metabolism through LDLR-mediated uptake and AMPK/mTOR pathway in the liver.

### Anlotinib induces lipid accumulation in human lung cancer cells

**Fig. 3:**
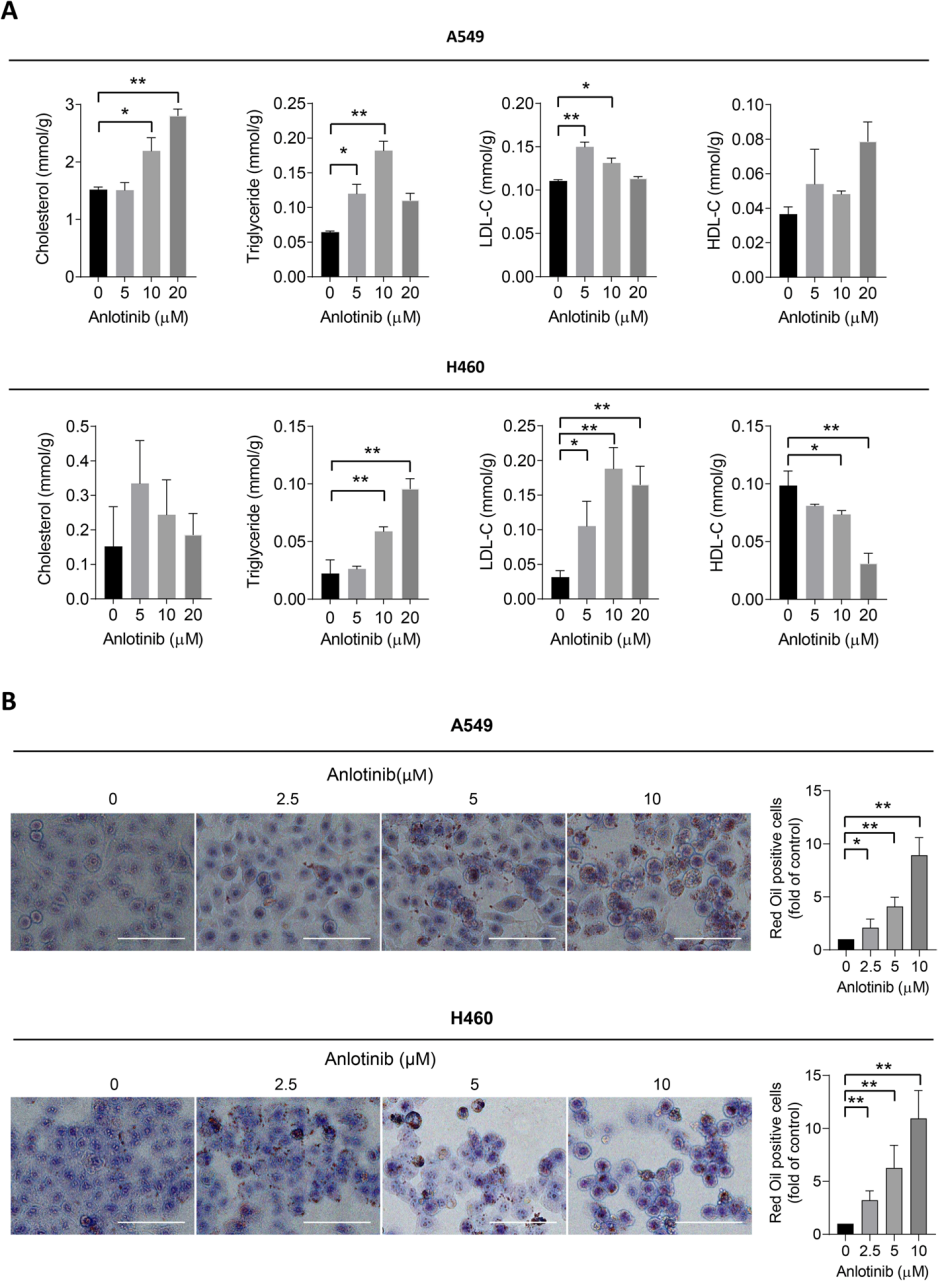
Anlotinib induces lipid accumulation in human lung cancer cells. **A** A549 and H460 cells were treated with various concentrations of anlotinib. Intracellular TC, TG, LDL-c and HDL-c levels were determined. **B** Oil Red O staining cells were photographed. Scale bar = 100 μm. *P < 0.05; **P < 0.01 by Student’s *t* test

Given that anlotinib is a promising small-molecule anticancer drug, verifying whether anlotinib controls lipid metabolism in cancer cells is crucial. In vitro experiments were carried out to examine the effects of anlotinib treatment on the lipid metabolism in cancer cells. Consistent with the findings from the mouse model, the intracellular concentrations of TC, TG, and LDL in A549 cells increased significantly as a result of anlotinib treatment. Moreover, H460 cells also exhibited a significant enhancement in intracellular TG and LDL levels, while the intracellular TC level showed a trend of increase (Fig. 3A). Additionally, Oil Red O staining revealed that cells treated with anlotinib exhibited significantly higher lipid accumulation in comparison to the control cells (Fig. 3B). These findings suggest that anlotinib influences lipid homeostasis in human lung cancer cells in addition to its anti-cancer effect.

### Anlotinib mediates key regulators and enzymes linked to lipid metabolism in cancer cells

**Fig. 4:**
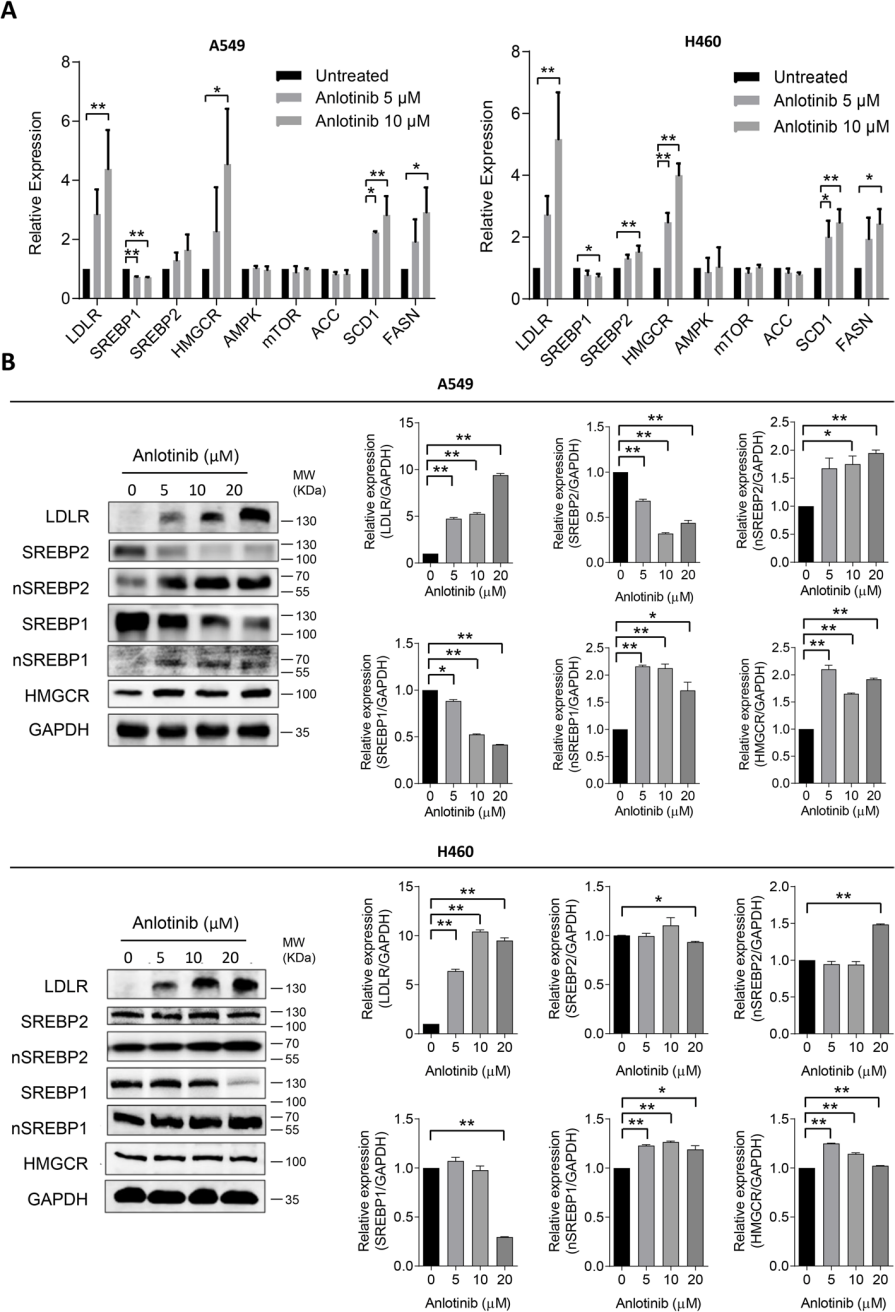
Anlotinib mediates key genes and enzymes required for cholesterol synthesis and uptake in human lung cancer cells. **A** A549 and H460 cells were treated with various concentrations of anlotinib. LDLR, SREBP1, SREBP2, HMGCR, AMPK, mTOR, ACC, SCD1 and FASN mRNA expression levels were determined. **B** LDLR, SREBP1, nuclear SREBP1 (nSREBP1), SREBP2, nuclear SREBP2 (nSREBP2) and HMGCR protein expression levels along with their respective quantitative histograms. *P < 0.05; **P < 0.01 by Student’s* t* test

The mRNA and protein levels of a number of transcriptional factors and enzymes involved in lipid synthesis and metabolism in human lung cancer cells were examined to further understand the impact of anlotinib treatment on these processes. As seen in Fig. 4A, after anlotinib treatment, there was a noticeable increase in the mRNA levels of LDLR, HMGCR, SCD1, and FASN in both A549 and H460 cells. SREBP1 mRNA levels, on the other hand, were markedly reduced in both cell types. The protein levels of LDLR consistently presented a considerable, dose-dependent rise. Moreover, there were obvious upward trends in the protein levels of nuclear SREBP1, nuclear SREBP2, and HMGCR (Fig. 4B). These findings suggest that anlotinib treatment modulates lipid synthesis and uptake processes in cancer cells.

### Anlotinib increases intracellular cholesterol levels in LDLR knockdown cells

**Fig. 5:**
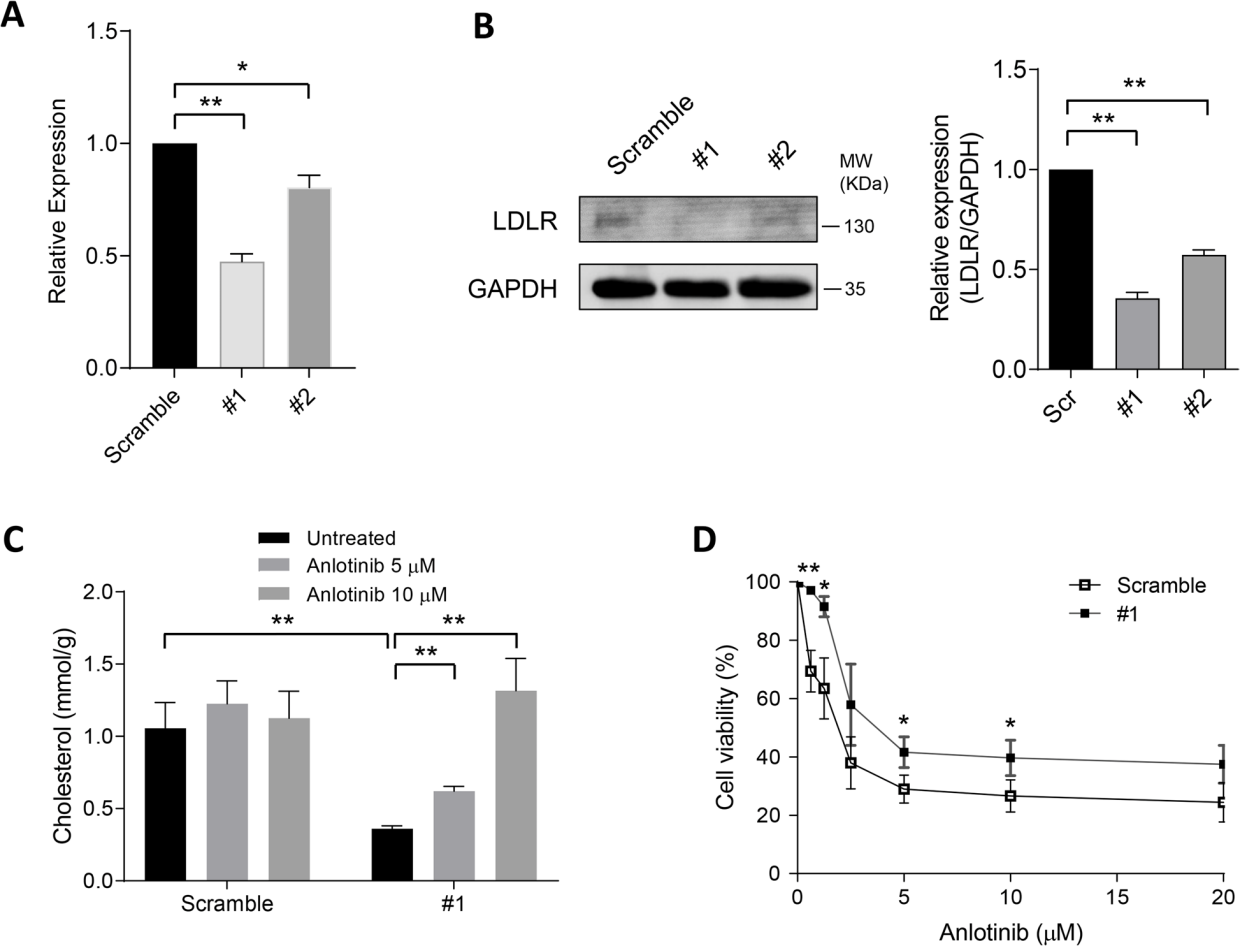
LDLR knockdown attenuates cancer cells sensitivity to anlotinib. **A** Quantitative PCR measurement of LDLR mRNA expression levels in H460 cells transfected with scramble or two separate pLKO.1-puro shRNA targeting LDLR sequences (#1 and #2). **B** LDLR protein expression levels were examined in scramble, #1 and #2 cells. **C** Intracellular TC level in scramble and #1 cells treated with different concentrations of anlotinib. **D** Different doses of anlotinib were administered to scramble and #1 cells during the course of 72 h. Viability was evaluated using metabolic MTT assay. *P < 0.05; **P < 0.01 by Student’s *t* test

Anlotinib treatment largely upregulated both mRNA and protein levels of LDLR. Then, the impact of LDLR knockdown on cholesterol homeostasis and the anticancer effect of anlotinib were investigated in human lung cancer cells. H460 cells expressing shRNA to knockdown LDLR was established. Both mRNA and protein levels of the knockdown efficiency were validated (Fig. 5A and B). Intracellular cholesterol levels were significantly decreased as a result of LDLR silencing (#1). However, the intracellular cholesterol content was noticeably raised following anlotinib treatment, even in LDLR-silenced cells. This suggests that LDLR knockdown promotes the activation of cholesterol synthesis as a compensatory mechanism to counteract the inefficient cholesterol uptake in the presence of anlotinib (Fig. 5C). Interestingly, LDLR silencing increased the proliferation rate of H460 cells (Fig. 5D). These findings suggest that LDLR may not be the primary regulator involved in anlotinib-induced elevated cholesterol levels. Additionally, LDLR knockdown decreased cell sensitivity to anlotinib, which was owing in part to an increase in intracellular cholesterol.

### LDL attenuates the efficacy of anlotinib in human lung cancer cells

**Fig. 6:**
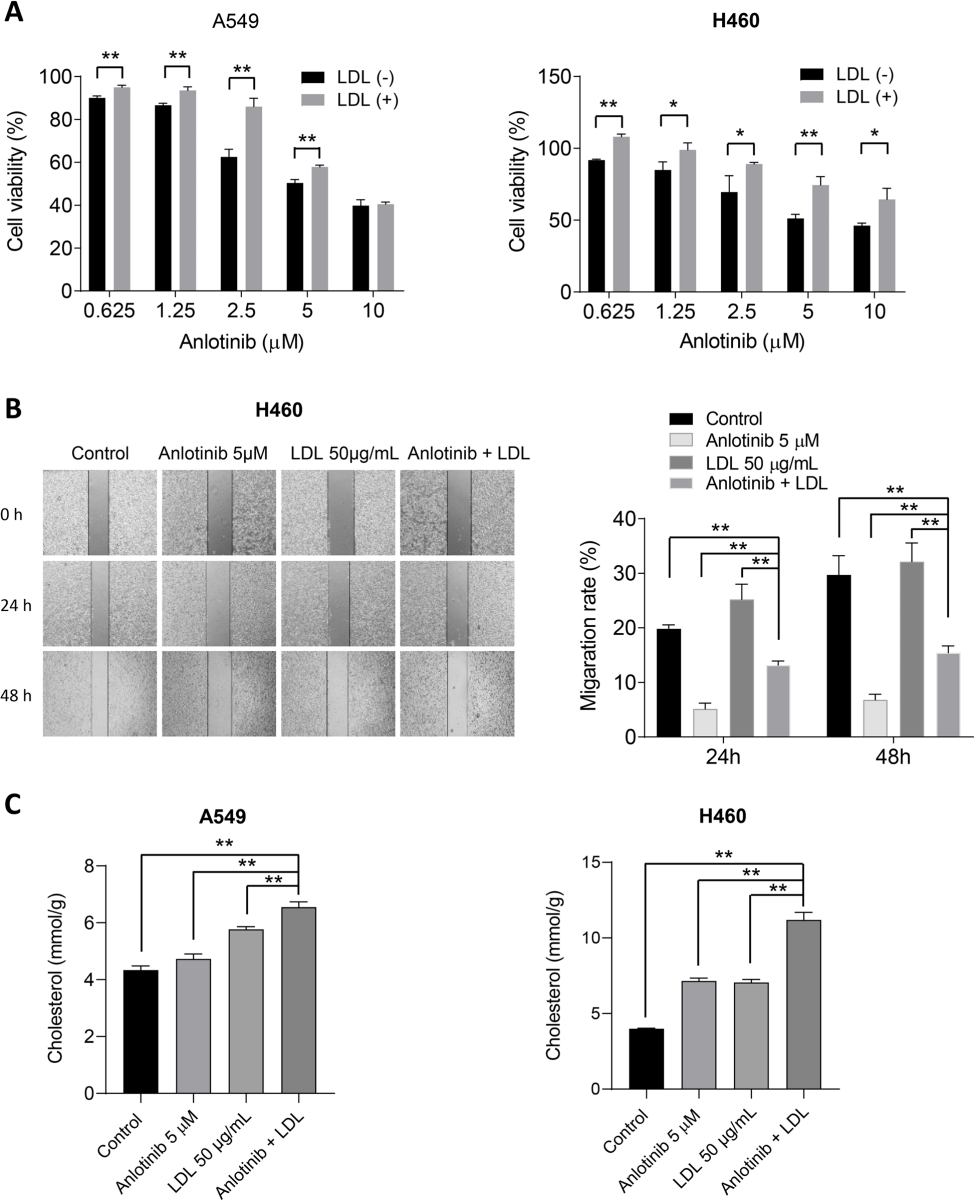
LDL dampens the efficacy of anlotinib. **A** Anlotinib alone or in combination with LDL (50 g/ml) was applied to A549 and H460 cells for 72 h. Viability was evaluated using metabolic MTT assay. **B** Wound-healing assay of H460 cells treated with anlotinib, LDL or both and their respective quantitative histograms. **C** Intracellular TC level in anlotinib treated cells. *P < 0.05; **P < 0.01 by Student’s *t* test

Furthermore, the impact of LDL addition on cell proliferation in anlotinib-treated A549 and H460 cells was investigated. The proliferation rate of cells treated with anlotinib was dramatically increased by the addition of LDL, as seen in Fig. 6A. Anlotinib alone and in combination with LDL had half-maximal inhibitory concentrations (IC_50_) in A549 cells of 5.6 μM and 7.2 μM, respectively. The IC_50_ values for anlotinib alone in H460 cells were 6.8 μM, while for the combination, they were 14.5 μM. Additionally, when anlotinib and LDL were used together, the effect of anlotinib on cell migration decreased in comparison to anlotinib alone (Fig. 6B). The levels of intracellular cholesterol were raised by anlotinib and LDL alone in both cell lines, but the combination had the strongest effect (Fig. 6C). These results indicate that elevated exogenous LDL reduces the cytotoxic effect of anlotinib.

## Discussion

Emerging evidence highlights the significance of lipid metabolism reprogramming as a hallmark of malignancy. To fulfill the increasing demand for macromolecular production and adapt to changing environment, cancer cells upregulate lipogenesis [19, 20]. However, the relationship between host lipid levels and tumorigenesis remains controversial. Reduced plasma levels of TC and LDL-c have been linked in some studies to poor survival in patients with various type of cancer [21, 22]. Conversely, other studies have suggested that hyperlipidemia can promote tumor growth and progression [3, 23, 24]. The underlying mechanisms governing this bidirectional interaction are still poorly understood and require further investigation. Over the last two decades, much emphasis has been paid to the metabolic consequences induced by targeted anti-cancer agents [25–28]. Hyperlipidemia is frequently observed in patients treated with VEGFR (vascular endothelial growth factor receptor)–TKIs [13], as well as mTOR inhibitors (such as rapamycin, temsirolimus and everolimus) [12]. It's interesting to note that hyperlipidemia has been suggested as a potential predictor of the efficacy of temsirolimus [29] and VEGFR-TKIs treatment [13] for mRCC. Similarly, hyperlipidemia has been found to be an independent predictor of the efficacy of anlotinib [6], suggesting that it may be a mechanism-based toxicity [30].

Lipid metabolism is a complex process regulated by multiple pathways. One key regulator of cellular metabolism and energy homeostasis is AMPK. AMPK functions as a direct upstream kinase of SREBPs. The transcriptional activity of SREBPs is suppressed by phosphorylated AMPK, which prevents lipogenesis in hepatocytes [31].

PI3K/Akt/mTOR signaling pathway [32] also plays a role in the regulation of SREBP-1 activation [32]. Previous studies have indicated that mTOR inhibitors can lead to a significant increase in plasma LDL-c levels by reducing hepatic LDLR expression [33, 34]. By facilitating the uptake and clearance of LDL particles, hepatic LDLR is a key factor in controlling plasma LDL-c levels. The intracellular cholesterol pool negatively regulates the transcriptional expression of LDLR, which is crucial for preserving cholesterol homeostasis. Studies by Huang JF et al. [24] have shown that reduced hepatic LDLR protein levels are associated with elevated levels of LDL-c in the blood and hyperlipidemia. Anlotinib significantly altered the lipid metabolism in C57BL/6 J mice in this study. Anlotinib treatment resulted in the downregulation of hepatic LDLR expression, which likely contributed to reduced LDL-c uptake and the development of hyperlipidemia. The upregulation of SREBP1 expression induced by anlotinib may be a result of decreased AMPK phosphorylation. Furthermore, both anlotinib and rapamycin exhibited significant inhibition of mTOR phosphorylation, implying that anlotinib partially affects lipid metabolism through the mTOR pathway. Therefore, anlotinib administration appears to influence cholesterol metabolism in the liver through LDLR-mediated uptake, as well as AMPK/mTOR pathways.

According to our findings, anlotinib regulates TC levels and key factors (such as SREBPs, LDLR, and HMGCR) of cholesterol metabolism in cancer cells. LDLR, which is highly expressed in various types of tumors, has been implicated in tumorigenesis [3, 35–37]. Studies have demonstrated that LDLR downregulation can reduce the growth of pancreatic cancer cells by preventing ERK1/2 signaling and improve the efficacy of chemotherapy [35]. Strikingly, anlotinib significantly increased intracellular TC even in LDLR knockdown cells, suggesting that downregulation of LDLR may promote intracellular cholesterol synthesis [38]. Furthermore, LDLR knockdown reduced the sensitivity of cells to anlotinib, showing that the cytotoxic effect of anlotinib is, at least in part, mediated by upregulation of LDLR, although the exact molecular mechanism remains unclear.

Moreover, exogenous LDL-c reduced cell sensitivity to anlotinib, indicating a possible function for cholesterol metabolism in controlling cancer cell sensitivity to anlotinib. Lipid metabolism alterations are known to be involved in resistance to conventional and targeted chemotherapies [39–41]. Previous studies have shown that exogenous LDL-c can attenuate the antitumor efficacy of TKIs in renal carcinoma cells, consistent with our findings [42]. Although some animal studies have reported worse efficacy of TKIs in hypercholesterolemic mice compared to those with normal cholesterol levels, these studies did not investigate changes in key regulators of the cholesterol pathway before and after TKIs treatment, nor their relationship with drug sensitivity. These results seemly contradict with the findings from clinical trials, which showed that anlotinib-associated hyperlipidemia was closely correlated with the favorable prognosis. It's possible that the conflicting findings are the result of intricate interactions among host lipids, lipid metabolism in the tumor microenvironment, and drug efficacy. Systemic lipid levels may not accurately reflect the lipid profile in cancer cells, and host lipids can impact drug efficacy by directly influencing tumor cells or modulating immune system functions [43, 44], which play a role in drug response. Understanding the intricate interplay between host lipids, tumor lipid metabolism, and drug efficacy will require further investigation. Future studies should clarify the meaning of lipid metabolism in the context of anlotinib treatment and investigate the particular molecular processes underlying the modulation of drug sensitivity by cholesterol metabolism.

### Strengths and limitations

The mechanisms of anltinib-induced lipid disorders are being examined for the first time in the present study. The results demonstrated that anotinib affected hepatic lipid metabolism, leading to the increased host lipid levels. Importantly, anlotinib significantly modulated the lipid homeostasis in cancer cells. Enhanced intracellular cholesterol attenuated the efficacy of anlotinib, suggesting that lipid metabolism has an impact in cancer cell sensitivity to anlotinib. Nevertheless, this study has still a few limitations. First, the mechanism by which anlotinib regulates lipid metabolism in the liver has not been validated in hepatocytes. Second, the C57BL/6 J mouse model was applied in in vivo experiments. Further studies will be designed to reveal the detailed mechanisms using mouse xenograft models. Then, a TKI control group was not included in in vitro experiments. At last, the impact of lipid-lowering medications on anlotinib efficacy was not investigated in this study.

## Conclusions

In summary, this study provides evidence that anlotinib modulates host lipid metabolism by multiple pathways. These effects could be attributed to hepatic LDLR downregulation as well as suppression of AMPK/mTOR signaling pathways. Furthermore, anlotinib exerts a significant impact on lipid metabolism in cancer cells by regulating key transcription factors and metabolic enzymes. These findings suggest a close association between anlotinib sensitivity and lipid metabolism in cancer cells, which sheds light on the relevance of lipid accumulation in anlotinib resistance and further motivation for improving clinical efficacy in patients treated with anlotinib-base therapy.

### Supplementary Information

**Additional file 1:** **Supplementary Table S1.** qRT-PCR primers for mouse.

**Additional file 2:** **Supplementary Table S2.** qRT-PCR primers for human.

## Data Availability

All data used and analyzed during this study will be available from the corresponding author upon reasonable request.

## Abbreviations

LDLR: Low-density lipoprotein receptor
LDL-c: Low-density lipoprotein cholesterol
LDL: Low-density lipoprotein
AMPK: AMP-activated protein kinase
mTOR: Mammalian target of rapamycin
SREBP: Sterol response element binding protein
VEGFR: Vascular endothelial growth factor receptor
PFS: Progress -free survival
OS: Overall survival
mRCC: Metastatic renal cell carcinoma
PI3K: Phosphoinositide 3-kinase
TKI: Tyrosine kinase inhibitor
LRP: LDL-related protein
TC: Cholesterol
TG: Triglyceride
HDL: High-density lipoprotein
HMGCR: 3-Hydroxy-3-methylglutaryl- coenzyme A reductase
DMEM: Dulbecco’s modified Eagle’s medium
FBS: Fetal bovine serum
MTT: 3- (4,5-Dimethylthiazol-2-yl)-2,5-diphenyltetrazolium bromide
DMSO: Dimethyl sulfoxide
PBS: Phosphate-buffered saline
BSA: Bovine serum albumin
H&E: Hematoxylin–Eosin
ACC1: Acetyl CoA carboxylase 1
FASN: Fatty acid synthase
SCD1: Stearoyl-CoA desaturase 1
IC_50_: Half-maximal inhibitory concentration

## References

[1] Nelson RH (2013). Hyperlipidemia as a risk factor for cardiovascular disease. Prim Care.

[2] O'Brien T, Nguyen TT, Zimmerman BR (1998). Hyperlipidemia and diabetes mellitus. Mayo Clin Proc.

[3] Gallagher EJ, Zelenko Z, Neel BA, Antoniou IM, Rajan L, Kase N (2017). Elevated tumor LDLR expression accelerates LDL cholesterol-mediated breast cancer growth in mouse models of hyperlipidemia. Oncogene.

[4] Mamtani R, Lewis JD, Scott FI, Ahmad T, Goldberg DS, Datta J (2016). Disentangling the Association between Statins, Cholesterol, and Colorectal Cancer: A Nested Case-Control Study. Plos Med.

[5] Han B, Li K, Wang Q, Zhang L, Shi J, Wang Z (2018). Effect of Anlotinib as a Third-Line or Further Treatment on Overall Survival of Patients With Advanced Non-Small Cell Lung Cancer: The ALTER 0303 Phase 3 Randomized Clinical Trial. Jama Oncol.

[6] Wang J, Zhao Y, Wang Q, Zhang L, Shi J, Wang Z (2018). Prognostic factors of refractory NSCLC patients receiving anlotinib hydrochloride as the third- or further-line treatment. Cancer Biol Med.

[7] Ma J, Song Y, Shou J, Bai Y, Li H, Xie X (2020). Anlotinib for patients with metastatic renal cell carcinoma previously treated with one vascular endothelial growth factor receptor-tyrosine kinase inhibitor: a phase 2 trial. Front Oncol.

[8] Zhou A, Bai Y, Song Y, Luo H, Ren X, Wang X (2019). Anlotinib versus sunitinib as first-line treatment for metastatic renal cell carcinoma: a randomized phase ii clinical trial. Oncologist.

[9] Houde VP, Brûlé S, Festuccia WT, Blanchard P, Bellmann K, Deshaies Y (2010). Chronic rapamycin treatment causes glucose intolerance and hyperlipidemia by upregulating hepatic gluconeogenesis and impairing lipid deposition in adipose tissue. Diabetes.

[10] Morrisett JD, Abdel-Fattah G, Hoogeveen R, Mitchell E, Ballantyne CM, Pownall HJ (2002). Effects of sirolimus on plasma lipids, lipoprotein levels, and fatty acid metabolism in renal transplant patients. J Lipid Res.

[11] Morrisett JD, Abdel-Fattah G, Kahan BD (2003). Sirolimus changes lipid concentrations and lipoprotein metabolism in kidney transplant recipients. Transplant Proc.

[12] Busaidy NL, Farooki A, Dowlati A, Perentesis JP, Dancey JE, Doyle LA (2012). Management of metabolic effects associated with anticancer agents targeting the PI3K-Akt-mTOR pathway. J Clin Oncol.

[13] Song Y, Du C, Zhang W, Sun Y, Yang L, Cui C (2016). A study on the association between hyperlipidemia and hypothyroidism and the response to TKIs in metastatic renal cell carcinoma. Asia-Pac J Clin Onco.

[14] Tassi R, Baldazzi V, Lapini A, Carini M, Mazzanti R (2015). Hyperlipidemia and hypothyroidism among metastatic renal cell carcinoma patients taking sunitinib malate. Related or unrelated adverse events?. Clin Genitourin Cancer.

[15] Baldazzi V, Tassi R, Lapini A, Carini M, Mazzanti R (2012). Newly onset hyperlipidemia in metastatic renal cell carcinoma patients treated with sunitinib. J Clin Oncol.

[16] Baldazzi V, Tassi R, Lapini A, Carini M, Mazzanti R (2011). Sunitinb-induced hyperlipidemia in patients with metastatic renal cell carcinoma. J Clin Oncol.

[17] Boucher P, Gotthardt M (2004). LRP and PDGF signaling: a pathway to atherosclerosis. Trends Cardiovasc Med.

[18] Loukinova E, Ranganathan S, Kuznetsov S, Gorlatova N, Migliorini MM, Loukinov D (2002). Platelet-derived growth factor (PDGF)-induced tyrosine phosphorylation of the low density lipoprotein receptor-related protein (LRP). Evidence for integrated co-receptor function betwenn LRP and the PDGF. J Biol Chem.

[19] Bian X, Liu R, Meng Y, Xing D, Xu D, Lu Z (2021). Lipid metabolism and cancer. J Exp Med.

[20] Broadfield LA, Pane AA, Talebi A, Swinnen JV, Fendt SM (2021). Lipid metabolism in cancer: New perspectives and emerging mechanisms. Dev Cell.

[21] Yang Z, Qin W, Chen Y, Yuan B, Song X, Wang B (2018). Cholesterol inhibits hepatocellular carcinoma invasion and metastasis by promoting CD44 localization in lipid rafts. Cancer Lett.

[22] Fiorenza AM, Branchi A, Sommariva D (2000). Serum lipoprotein profile in patients with cancer. A comparison with non-cancer subjects. Int J Clin Lab Res.

[23] Wang C, Li P, Xuan J, Zhu C, Liu J, Shan L (2017). Cholesterol enhances colorectal cancer progression via ROS elevation and MAPK signaling pathway activation. Cell Physiol Biochem.

[24] Huang J, Li L, Lian J, Schauer S, Vesely PW, Kratky D (2016). Tumor-induced hyperlipidemia contributes to tumor growth. Cell Rep.

[25] Vergès B, Walter T, Cariou B (2014). Endocrine side effects of anti-cancer drugs: effects of anti-cancer targeted therapies on lipid and glucose metabolism. Eur J Endocrinol.

[26] Bhatnagar R, Dixit NM, Yang EH, Sallam T (2022). Cancer therapy’s impact on lipid metabolism: Mechanisms and future avenues. Front Cardiovasc Med.

[27] Puliani G, Appetecchia M (2021). Endocrine toxicities of antineoplastic therapy. Cancers.

[28] Breccia M, Molica M, Alimena G (2014). How tyrosine kinase inhibitors impair metabolism and endocrine system function: A systematic updated review. Leukemia Res.

[29] Lee CK, Marschner IC, Simes RJ, Voysey M, Egleston B, Hudes G (2012). Increase in Cholesterol Predicts Survival Advantage in Renal Cell Carcinoma Patients Treated with Temsirolimus. Clin Cancer Res.

[30] Cho DC, Atkins MB (2012). Serum Cholesterol and mTOR Inhibitors: Surrogate Biomarker or Epiphenomenon?. Clin Cancer Res.

[31] Li Y, Xu S, Mihaylova MM, Zheng B, Hou X, Jiang B (2011). AMPK phosphorylates and inhibits SREBP activity to attenuate hepatic steatosis and atherosclerosis in diet-induced insulin-resistant mice. Cell Metab.

[32] Bakan I, Laplante M (2012). Connecting mTORC1 signaling to SREBP-1 activation. Curr Opin Lipidol.

[33] Ai D, Chen C, Han S, Ganda A, Murphy AJ, Haeusler R (2012). Regulation of hepatic LDL receptors by mTORC1 and PCSK9 in mice. J Clin Invest.

[34] Ma KL, Ruan XZ, Powis SH, Chen Y, Moorhead JF, Varghese Z (2007). Sirolimus Modifies Cholesterol Homeostasis in Hepatic Cells: A Potential Molecular Mechanism for Sirolimus-Associated Dyslipidemia. Transplantation.

[35] Guillaumond F, Bidaut G, Ouaissi M, Servais S, Gouirand V, Olivares O (2015). Cholesterol uptake disruption, in association with chemotherapy, is a promising combined metabolic therapy for pancreatic adenocarcinoma. Proc Natl Acad Sci U S A.

[36] Guo D, Reinitz F, Youssef M, Hong C, Nathanson D, Akhavan D (2011). An LXR agonist promotes glioblastoma cell death through inhibition of an EGFR/AKT/SREBP-1/LDLR-dependent pathway. Cancer Discov.

[37] Yue S, Li J, Lee SY, Lee HJ, Shao T, Song B (2014). Cholesteryl ester accumulation induced by PTEN loss and PI3K/AKT activation underlies human prostate cancer aggressiveness. Cell Metab.

[38] Chen Z, Chen L, Sun B, Liu D, He Y, Qi L (2021). LDLR inhibition promotes hepatocellular carcinoma proliferation and metastasis by elevating intracellular cholesterol synthesis through the MEK/ERK signaling pathway. Mol Metab.

[39] Germain N, Dhayer M, Boileau M, Fovez Q, Kluza J, Marchetti P (2020). Lipid metabolism and resistance to anticancer treatment. Biology.

[40] Giacomini I, Gianfanti F, Desbats MA, Orso G, Berretta M, Prayer-Galetti T (2021). cholesterol metabolic reprogramming in cancer and its pharmacological modulation as therapeutic strategy. Front Oncol.

[41] Kopecka J, Trouillas P, Gašparović A, Gazzano E, Assaraf YG, Riganti C (2020). Phospholipids and cholesterol: Inducers of cancer multidrug resistance and therapeutic targets. Drug Resist Update.

[42] Naito S, Makhov P, Astsaturov I, Golovine K, Tulin A, Kutikov A (2017). LDL cholesterol counteracts the antitumour effect of tyrosine kinase inhibitors against renal cell carcinoma. Brit J Cancer.

[43] Qin WH, Yang ZS, Li M, Chen Y, Zhao XF, Qin YY (2020). High serum levels of cholesterol increase antitumor functions of nature killer cells and reduce growth of liver tumors in mice. Gastroenterology.

[44] Yang W, Bai Y, Xiong Y, Zhang J, Chen S, Zheng X (2016). Potentiating the antitumour response of CD8+ T cells by modulating cholesterol metabolism. Nature.

