# Supplementary figures and images for: Anlotinib affects systemic lipid metabolism and induces lipid accumulation in human lung cancer cells
Source: Lipids Health Dis. 2023 Aug 23;22:134. doi: 10.1186/s12944-023-01907-y (PMC10464365; doi:10.1186/s12944-023-01907-y)

Table S1. qRT-PCR primers for mouse.

**
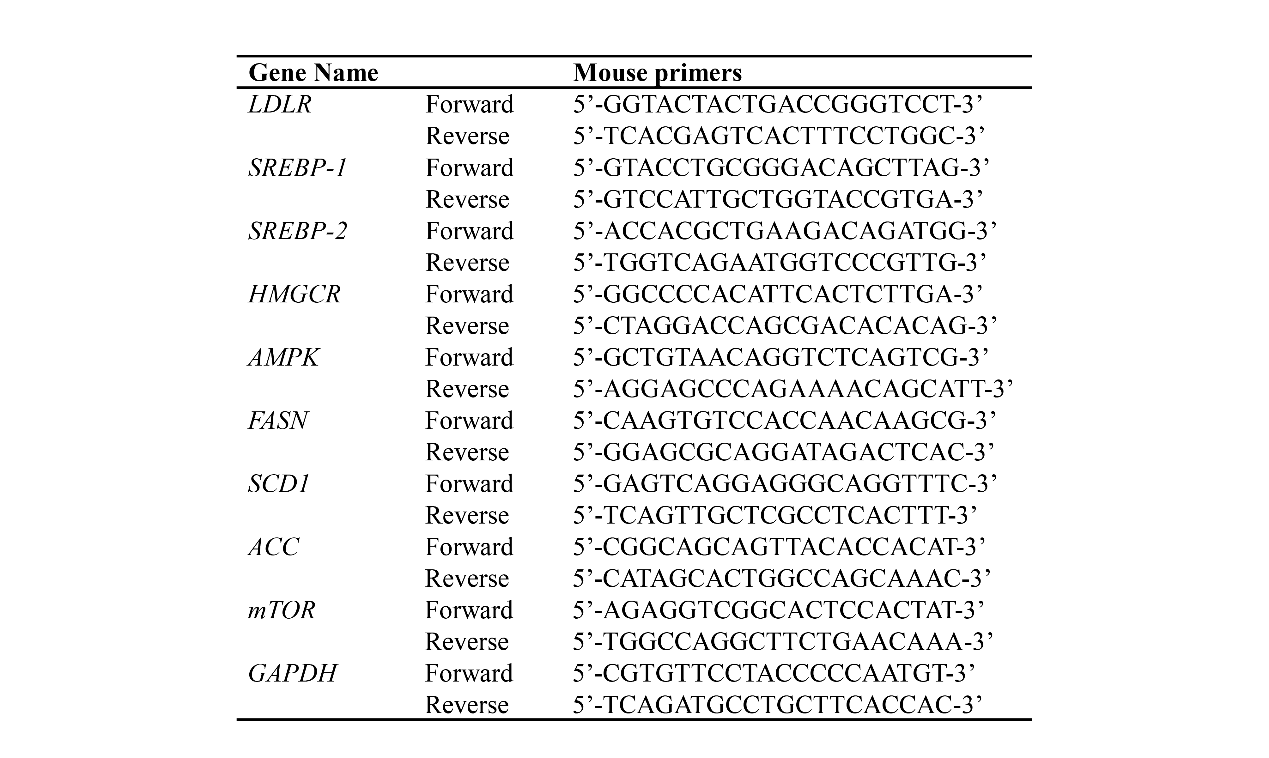
**

Supplement: Supplementary file 1 — Additional file 1: Supplementary Table S1. qRT-PCR primers for mouse. [file 12944_2023_1907_MOESM1_ESM.docx]

Table S2. qRT-PCR primers for human.


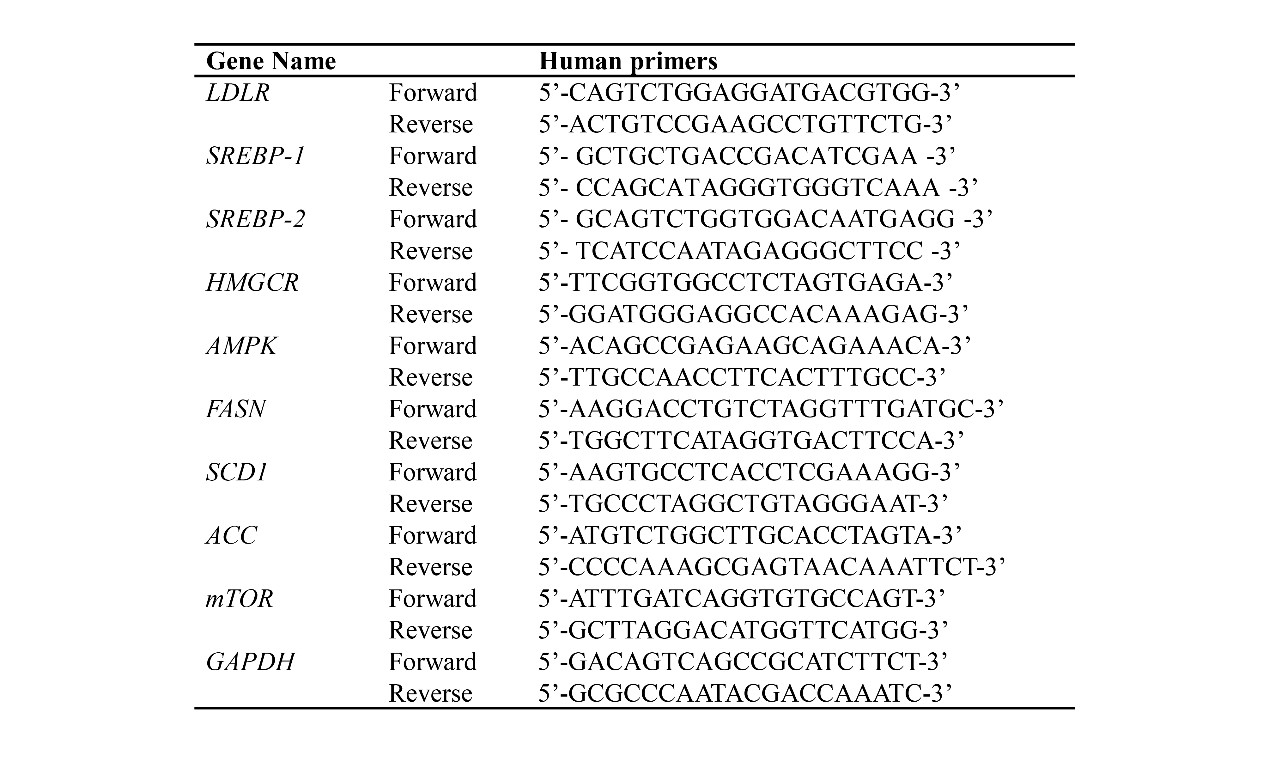

Supplement: Supplementary file 2 — Additional file 2: Supplementary Table S2. qRT-PCR primers for human. [file 12944_2023_1907_MOESM2_ESM.docx]
